# Pulmonary Biomarkers Based on Alterations in Protein Expression after Exposure to Arsenic

**DOI:** 10.1289/ehp.9611

**Published:** 2007-01-08

**Authors:** R. Clark Lantz, Brandon J. Lynch, Scott Boitano, Gerald S. Poplin, Sally Littau, George Tsaprailis, Jefferey L. Burgess

**Affiliations:** 1 Department of Cell Biology & Anatomy; 2 Southwest Environmental Health Science Center; 3 Department of Physiology; 4 Arizona Respiratory Center; 5 Division of Community, Environment and Policy and; 6 Center for Toxicology, University of Arizona, Tucson, Arizona, USA

**Keywords:** arsenic, human, lavage, lung, mice, proteomics, sputum

## Abstract

**Objective:**

Environmental exposure to arsenic results in multiple adverse effects in the lung. Our objective was to identify potential pulmonary protein biomarkers in the lung-lining fluid of mice chronically exposed to low-dose As and to validate these protein changes in human populations exposed to As.

**Methods:**

Mice were administered 10 or 50 ppb As (sodium arsenite) in their drinking water for 4 weeks. Proteins in the lung-lining fluid were identified using two-dimensional gel electrophoresis (*n* = 3) or multidimensional protein identification technology (MUDPIT) (*n* = 2) coupled with mass spectrometry. Lung-induced sputum samples were collected from 57 individuals (tap water As ranged from ~ 5 to 20 ppb). Protein levels in sputum were determined by ELISA, and As species were analyzed in first morning void urine.

**Results:**

Proteins in mouse lung-lining fluid whose expression was consistently altered by As included glutathione-*S*-transferase (GST)-omega-1, contraspin, apolipoprotein A-I and A-IV, enolase-1, peroxiredoxin-6, and receptor for advanced glycation end products (RAGE). Validation of the putative biomarkers was carried out by evaluating As-induced alterations in RAGE in humans. Regression analysis demonstrated a significant negative correlation (*p* = 0.016) between sputum levels of RAGE and total urinary inorganic As, similar to results seen in our animal model.

**Conclusion:**

Combinations of proteomic analyses of animal models followed by specific analysis of human samples provide an unbiased determination of important, previously unidentified putative biomarkers that may be related to human disease.

Estimation of health risks associated with environmental exposure to low doses of toxicants is essential to improving human health risk assessment. As such, the Commission on Risk Assessment and Risk Management of the National Research Council (NRC 1997) has recommended development of environmental research and management tools. The science made possible through proteomic analysis provides a useful investigative tool to accomplish these recommendations through simultaneous analysis of numerous proteins in one sample. Through this approach, it is possible to detect molecular changes associated with low-dose environmental exposure before the development of clinically apparent disease. By using patterns of changes in these proteins measured simultaneously, it is also possible to deduce mechanisms of action in the pathologic development of disease. These mechanisms can be compared between different toxicants and biological systems, including humans, human cell lines, and rodents.

One toxicant that is both relevant and far reaching in human exposure is arsenic. Arsenic is a naturally occurring metalloid found in water, soil, and air, and exposure to inorganic As occurs through both environmental and occupational sources (International Agency for Research on Cancer 1987). Epidemiologic studies have associated As ingestion with an increased risk in many human cancers, including lung, skin, bladder, kidney, liver, and stomach (Chiou et al. 1995; Wu et al. 1989). Arsenic is also associated with non-malignant diseases such as peripheral vascular disease (Chiou et al. 2005), diabetes (Navas-Acien et al. 2006), and chronic lung disease (Smith et al. 2006). Although chronic exposure to moderate and/or high levels of As in drinking water may lead to the development of cancer in humans, the effects of lower, environmentally relevant doses are only inferred through models of high exposure. There is little data evaluating the molecular effect of chronic, low-dose As exposure. This is partly due to the absence of measurable end points in this process.

The molecular and cellular mechanisms by which As acts as a toxicant have not been clearly elucidated, although as a carcinogen, it appears to act through epigenetic mechanisms. Arsenic has been implicated in promoting alterations in multiple cellular pathways, including growth and proliferation pathways, apoptotic pathways, DNA repair mechanisms, immunosurveillance, and stress–response pathways (Rossman 2003).

In As-exposed experimental systems, alterations accompanying transformation to the malignant phenotype have been defined utilizing cDNA microarray technology to characterize gene expression (Chen et al. 2001; Lu et al. 2001). Altered genes implicated a number of cell mechanistic pathways including cell-cycle regulation, signal transduction, stress response, and apoptosis, as well as cytokine, growth factor, and hormone receptor genes.

Microarray analysis has been used for global determination of As-induced alterations, but the relationship between transcripts and the proteome is not easily transferred (Chen et al. 2003). Because it is ultimately the change in protein expression that alters the cellular response, evaluation of the proteome may allow for a more direct measure of As effects. Methods for analyzing multiple proteins have greatly advanced in the last several years with the ability of mass spectrometry (MS) and tandem MS (MS/MS) to analyze peptides extracted following protein isolation using two-dimensional (2-D) gel electrophoresis and proteolytic digestion. These techniques have the advantage that proteins are much more stable than mRNA obtained from human populations and that post-translational modification of selected proteins can be detected using MS-based proteomic techniques. In addition, lung-lining fluid may provide a source to detect lung-specific changes in protein response to toxicant exposures. Use of 2-D gel electrophoresis to examine alterations in protein expression in mouse epithelial lining fluid in ozone-sensitive and ozone-resistant mice has been reported (Wattiez et al. 2003). Previous proteomics studies involving As have relied on *in vitro* studies of single cell cultures (He et al. 2003; Lau et al. 2004). To our knowledge, no such data exist for As-induced changes in expression of proteins in lung-lining fluid.

In this article, we report As-induced changes in lung-lining fluid proteins of mice that occur after chronic, low-level exposures. To date, data obtained from animal research has been used to validate one of the potential protein biomarkers in human populations exposed to As.

## Materials and Methods

All chemicals and reagents were purchased from Sigma Chemical Company (St. Louis, MO) unless otherwise noted.

### Animal exposure

Male C57B16 mice from Jackson Laboratories (Bar Harbor, ME), 21 days of age, were exposed to As through drinking water for a total of 4 weeks. A total of 15 animals were split into three exposure groups: 50 ppb As, 10 ppb As, and a control group (water only, pH 7.0). Water for the 50-ppb and 10-ppb As treatment groups was prepared from sodium arsenite and the pH was adjusted to 7.0 with hydrochloric acid and sodium hydroxide. Water was administered to the animals 3 days weekly (Monday, Wednesday, Friday). Concentrations of As, were validated by inductively coupled plasma (ICP)-MS analysis. All animals were treated humanely and with regard for alleviation of suffering. All protocols were approved by the institutional animal care and use committee.

### Bronchoalveolar lavage

At the end of the 4-week exposure, animals were sacrificed by carbon dioxide exposure, and bronchoalveolar lavage (BAL) fluid (BALF) was collected as described by Wattiez et al. (2003).

### Acetone precipitation

Proteins were precipitated overnight in an 80% acetone solution prepared with HPLC grade acetone. The solution was then centrifuged for 10 min (5 × *g* at 4°C) to remove protein from the solution. The pellet was rinsed twice with 3 mL HPLC grade acetone at 4°C for 5 min each wash cycle (5 × *g* at 4°C).

### Determination of protein concentration

Protein concentrations were determined using the Coomassie Plus Assay Kit (Pierce, Rockford, IL). Bovine serum albumin was used as the standard. Samples were diluted in 200-μL aliquots of ultra-pure water before protein cleanup.

### Protein cleanup

We split each 200-μL sample into two 100-μL cleanup volumes. Samples were purified using a Bio-Rad ReadyPrep 2-D Cleanup Kit (163-2160; Bio-Rad, Hercules, CA) following the manufacturer’s instructions. After cleanup was complete, samples were rehydrated with Bio-Rad ReadyPrep 2-D Rehydration/Sample Buffer 1 (7 M urea, 2 M thiourea, 1% amido-sulfobetaine-14, 40 mM Tris). Samples were diluted to a 500 μg/mL concentration for analysis by the Southwest Environmental Health Science Center/Arizona Cancer Center Proteomics Core.

### Western blots

Mouse BAL samples were suspended in Laemmli buffer and run on 12.5% SDS-PAGE. Samples were transferred to nitrocellulose and blotted with anti-RAGE (receptor for advanced glycation end products) antibody (R&D Systems, Minneapolis, MN) followed by horseradish peroxidase–linked secondary antibody (Sigma). Blots were developed with the SuperSignal West Femto kit (Pierce). We determined band density using the Chemidoc XRS system under Quantity-one software control (Bio-Rad).

### 2-D gel electrophoresis

We submitted three samples from each exposure group (200 μL containing 100 μg protein) for 2-D SDS-PAGE separation. Each sample was run on an 11-cm immobilized pH gradient strip, pH 5–8, and then resolved on 12.5% Tris-HCl gel. Gels were stained using silver solution (2.5% wt/vol silver nitrate and 37% wt/vol formaldehyde) for 20 min.

### Image acquisition and analysis

Stained gels were imaged using an Investigator ProPic and HT Analyzer software, both from Genomic Solutions (Ann Arbor, MI). Intensity was calibrated with an intensity step wedge before the image was taken, and spot intensity was normalized with background subtraction. Gels were overlaid using Phoretix 2-D Expression software (Nonlinear Dynamics, Durham, NC). Gels for controls and treated groups were superimposed and stretched to match corresponding spots on each gel. Spots that varied by intensity, appeared to be shifted, or were absent from either group were picked robotically and saved for mass spectrometry identification.

### Liquid chromatography (LC)-MS/MS analysis

Excised protein spots were destained in a 1:1 mixture of 30 mM potassium ferro-cyanide and 100 mM sodium thiosulfate. Samples were digested in trypsin (10 μg/mL) overnight at 37°C. LC-MS/MS analyses were carried out using a quadrupole ion trap ThermoFinnigan LCQ DECA XP PLUS (ThermoFinnigan, San Jose, CA) equipped with a Michrom Paradigm MS4 HPLC (Michrom Biosources, Auburn, CA) and a nanospray source. Peptides were eluted from a 15-cm pulled tip capillary column (100 μm i.d. × 360 μm o.d; 3–5 μm tip opening) packed with 7 cm Vydac C18 (Vydac, Hesperia, CA) material (5 μm, 300Å pore size), using a gradient of 0–65% solvent B (98% methanol/2% water/0.5% formic acid/0.01% triflouroacetic acid) over a 60-min period at a flow rate of 350 nL/min. The electrospray ionization–positive mode spray voltage was set at 1.6 kV, and the capillary temperature was set at 200°C. We performed data-dependent scanning using Xcalibur, version 1.3, software (ThermoFinnigan) as described by Andon et al. (2002); we used a default charge of 2, an isolation width of 1.5 amu, an activation amplitude of 35%, an activation time of 30 msec, and a minimal signal of 10,000 ion counts. Global dependent data settings were as follows: reject mass width, 1.5 amu; dynamic exclusion enabled; exclusion mass width, 1.5 amu; repeat count, 1; repeat duration, 1 min; and exclusion duration, 5 min. Scan event series included one full scan with mass range 350–2,000 Da, followed by three dependent MS/MS scans of the most intense ion. MS/MS spectra were analyzed with Turbo SEQUEST, version 3.1 (ThermoFinnigan), a program that allows the correlation of experimental MS/MS data with theoretical spectra generated from known protein sequences. The peak list for the search was generated by Xcalibur 1.3. Parent peptide mass error tolerance was set at 1.5 amu, and fragment ion mass tolerance was set at 0.5 amu during the search. All spectra were searched against the latest version of the non-redundant protein database downloaded 14 July 2005 from the National Center for Biotechnology Information (NCBI 2005). We used X!Tandem (Global Proteome Machine Organization, Vancouver, British Columbia, Canada), to validate protein identification and as a means of highlighting false positive protein identifications by searching the data with a “reversed” version of the protein database (Craig et al. 2004). Proteins found during the reversed search were subsequently removed from the protein list.

### MUDPIT analysis

We analyzed two additional samples from each exposure group by multidimensional protein identification technology (MUDPIT). For these complex protein samples, ammonium bicarbonate was added to a concentration of 0.1 M; 40 μL of 10 mM DTT was then added before reducing at 56°C for 45 min (Wolters et al. 2001). Reduced cysteines were alkylated by addition of 40 μL of 55 mM iodoacetamide (10 mM final concentration) and incubated for 30 min at room temperature. Proteolysis was initiated with a 1:25 ratio (by weight) of sequencing grade modified trypsin and allowed to proceed overnight at 37°C. The digest was stored at −20°C until analysis.

For MUDPIT, we used a microbore HPLC system (Paradigm MS4; Michrom, Auburn, CA) with two separate strong cation exchange and reversed phase columns: a 100 μm I.D. capillary packed with 7 cm of 5 μm Vydac C18 reversed phase resin and a separate 250 μm I.D. capillary packed with 7 cm of 5 μm Partisphere strong cation exchanger resin (Whatman, Clifton, NJ). The sample (4.36 μg) was acidified using trifluoroacetic acid (TFA) and manually injected onto the strong cation exchange column, the effluent from the column being fed through reversed phase column. A representative 12-step MUDPIT analysis includes the following solutions: 10% methanol/0.1% formic acid, 0.01%TFA (buffer A); 95% methanol/0.1% formic acid, 0.01% TFA (buffer B); 10% methanol/0.1% formic acid, 0.01% TFA (buffer C); and 500 mM ammonium acetate/10% methanol/0.1% formic acid, 0.01% TFA (buffer D). Step 1 consisted of a 5-min equilibration step at 100% buffer A, another equilibration step for 5 min at 25% buffer B (75% buffer A), and a 40 min gradient from 25% buffer B to 65% buffer B, followed by 10 min 65% buffer B and 10 min 100% buffer A. Chromatography steps 2–12 followed the same pattern: 15 min of the appropriate percentage of buffers C and D followed by a 2-min 100% buffer C wash, a 5-min wash with 100% buffer A, equilibration with 25% buffer B for 5 min, a gradient from 25% buffer B to 65% buffer B in 40 min; and finally a 10-min 65% buffer B wash and a 10-min 100% buffer A wash. The buffer C/buffer D percentages used were 95/5%, 90/10%, 85/15%, 80/20%, 70/30%, 60/40%, 40/60%, 20/80%, 0/100%, 0/100%, and 0/100% for the 11 salt steps. MS/MS and database searching conditions were the same as those described above (Andon et al. 2002). We used DTASelect (Tabb et al. 2003) to filter the results according to previous criteria for reliable sequence peptides (Hunter et al. 2002; Wolters et al. 2001).

### Human study

This study was approved by the institutional review board at the University of Arizona. Study methods have been reported elsewhere (Josyula et al. 2006). Briefly, household recruitment took place between June 2002 and August 2003 in Ajo, Arizona (tap water As, ~ 20 ppb), and Tucson, Arizona (tap water As, ~ 5 ppb). Inclusion criteria required at least 3 years continuous residence at the time of recruitment, use of tap water for drinking and food preparation, and no current smoking. Information related to demographics, medical history, and occupational and environmental As exposures was collected. Information on consumption of seafood and/or mushrooms during the previous 3-day period was collected to assess other potential sources of As exposure.

### Sample collection

A first morning void urine sample was collected in two sterile 120-mL screw-topped polypropylene containers, which were previously tested and determined to be As free. Urine samples were processed within 2 hr of collection. For each household, tap water from the cold water faucet in the kitchen was allowed to run for 1 min before collecting water into two sterile 50-mL conical vials. Samples were stored at 4°C until transported to the University of Arizona for As analysis. An HPLC-ICP-MS speciation method was modified for the measurement of As (Gong et al. 2001). Urinary total inorganic As was defined as the sum of arsenite (As^3+^), arsenate (As^5+^), monomethylarsonic acid (MMA^V^), and dimethylarsinic acid (DMA).

Sputum induction was performed as previously described by Burgess et al. (2002). Subjects were asked to breathe a sterile 3% saline aerosol (Baxter, Deerfield, IL) generated using DeVilbiss Ultra-Neb 99HD ultrasonic nebulizers (Sunrise Medical, Somerset, PA) set on maximum output. All sputum supernatant samples were analyzed in duplicate for levels of RAGE using commercially available ELISA (R&D Systems).

### Statistical analysis

We used SPSS 12.0 (SPSS, Chicago, IL) and Stata 8.2 (StataCorp, College Station, TX) software for statistical analyses. For parametric tests, sputum RAGE concentrations were normalized using natural log transformations. Independent-sample *t*-tests were used to compare population characteristics by town, and RAGE concentrations by sex, race, town, diabetes, ever-asthma, and past smoking. Pearson’s correlation analyses were used to assess correlations among the different sputum biomarkers. We used multiple linear regression models to assess the association of urinary As and sputum RAGE, adjusting for potential confounders. For each of the outcome measures, variables found to be marginally significant in univariate regression analyses were included in a stepwise regression model using a backward elimination technique to decipher final models. Diagnostics were then performed for each model to assess whether assumption parameters for linear regression were met. To account for possible confounding effects, we included town, ever-asthma and MMA^V^/(As^3+^+As^5+^) in each model, regardless of their statistical significance. Town was forced into the models to account for other environmental or biological differences not assessed in this study. Similarly, asthma was included to account and adjust for known lung inflammation associated with the disease, and the ratio of MMA^V^/(As^3+^+As^5+^) was used as a measure of active methylation of inorganic As.

## Results

We used 2-D SDS-PAGE/isoelectric focusing separation of BALproteins from controls (*n* = 3) and animals treated with 10 ppb (*n* = 3) and 50 ppb As (*n* = 3) to identify protein expression that was consistently altered by As. To date, 25 spots have been analyzed (12 from animals treated with 50 ppb As and 13 from controls). Only proteins in which multiple peptides were identified were considered positively identified proteins. Identities of the proteins of interest from the spots selected for analysis are shown in Table 1. Identified proteins with expression that decreased with As exposure (higher intensity in the control gel or absent in the As-treated gel) were RAGE, glutathione-*S*-transferase (GST)-omega-1, contraspin (a serine—or cysteine—proteinase inhibitor isoform), apolipoprotein A-IV, and apolipoprotein A-I. Arsenic induced increased expression of both peroxiredoxin-6 and enolase-1.

Lavage proteins from two additional animals in each group were subjected to MUDPIT analysis. Only proteins in which multiple peptides were identified or in which single peptides were identified in multiple animals were considered positively identified proteins. Albumin and hemoglobins were excluded. Using these criteria, a total of 44 proteins were identified (Table 2). Six of the seven proteins identified using the 2-D gels were also present in the MUDPIT analysis (Table 2). Identification of these proteins by two distinct proteomic analyses makes them excellent candidates for dose–response quantitative analysis.

We selected RAGE for further analysis and validation because of its association with chronic inflammatory diseases and the availability of commercial reagents. Data from 2-D gels suggested that As exposure resulted in a decrease in BALF RAGE levels in mice. Western blots verified this conclusion. Blots from the same two animals used in MUDPIT analysis showed a dose-dependent decrease in band density of RAGE for samples exposed to 0, 10, and 50 ppb As (data not shown).

Although animal models are useful for discovery of potential biomarkers, these bio-markers must be validated and tested in human populations exposed to As. To evaluate the effect of As using lung-specific, minimally invasive techniques, we chose to determine the effect of As on levels of RAGE in human induced sputum.

The tested subjects were part of a larger study of 73 individuals who met inclusion criteria (Josyula et al. 2006): 40 from 33 households in Ajo and 33 from 30 households in Tucson. Of these subjects, 57 (78%) provided sputum, and there were no significant differences in demographics, smoking history, asthma, or diabetes between subjects who did (*n* = 57) and did not (*n* = 16) provide sputum samples. Of the 57 subjects providing sputum, all had adequate volumes for RAGE analysis. This population of 57 subjects ranged in age from 30 to 92 years, and was predominantly female, over 66% non-Hispanic white, and college educated (Table 3). The Tucson and Ajo populations did not differ significantly in age distribution, sex, race, asthma or diabetes diagnoses, or past smoking history.

**Figure 1:**
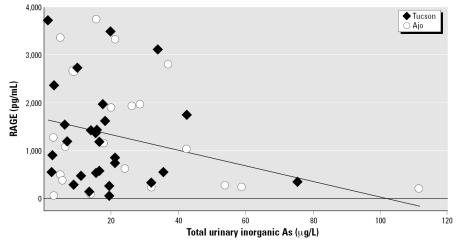
Sputum RAGE concentrations determined using ELISA techniques and plotted as a function of total urinary As levels. Because of the overlap in urinary As, data from all individuals from both Ajo and Tucson are plotted; outliers have been excluded. The regression line is from analysis detailed in Table 4 and shows a decrease in sputum RAGE as a function of urinary As.

Sputum RAGE concentrations were not significantly different by town (Table 3). However, there was a marked overlap of urinary total inorganic As concentrations across the towns. Therefore, we compared sputum RAGE concentrations with urinary total inorganic As concentration, combining both towns, as shown in Figure 1. Six individual concentrations of RAGE were identified as outliers using the method of Hadi (1992) for multivariate data; five of these were below detectable levels. Regression analyses were conducted without and with these observations. Table 4 displays results from regression analyses excluding the defined outliers. We found a significant negative association between urinary total inorganic As concentrations and RAGE expression. Urinary total inorganic As was not significant when the outliers were included; however, the pentavalent form of As in urine (As^5+^) was found to be significant in this setting (β = −0.304; *p* = 0.026). In addition, with the outliers included, having diabetes became significantly associated with sputum RAGE (β = 1.93; *p* = 0.021), and the contribution of body mass index (BMI) remained statistically significant. Asthma and the extent of methylation of As were not found to be associated with RAGE in either model. Town was kept in the model to account for potential differences in environmental exposures between communities that were not measured in this study, even though it was not statistically significant.

To test whether As compounds would directly bind to the receptor and interfere with quantitative ELISA measurements, we added 50 ppb sodium arsenite or 5 ppb monomethylarsonous acid (MMA^III^) to standards containing recombinant RAGE protein. Arsenic addition did not interfere with the measurement of RAGE levels (data not shown). To evaluate whether ligands or proteases in the induced sputum were affecting the levels of measured RAGE, recombinant RAGE was added to two sputum samples. No differences were seen when these samples were compared with RAGE standards (data not shown).

## Discussion

Discovery of biomarkers responsive to low-dose environmental exposures will greatly improve human health risk assessment. Using a proteomics approach, we have used data collected from animal models to drive evaluation and validation in human protein samples collected using minimally invasive techniques. Several proteins were consistently altered in 2-D gels from mouse BALF. Six of these proteins were also identified in MUDPIT analysis, providing additional confidence that they may be important biomarkers of As exposure and effect. We subjected one of these proteins, RAGE, to further analysis. Western blot analysis indicated an As-induced decrease in RAGE in mouse BALF. Using quantitative ELISA analysis, we verified that RAGE levels are also altered as a function of As levels in human populations. We have not yet analyzed the other potential biomarkers in human samples.

RAGE is a multiligand member of the immunoglobulin superfamily of cell surface receptors (Bierhaus et al. 2005). In addition to being a receptor for products of glycation and oxidation of proteins and lipids, RAGE has more recently been shown to bind other pro-inflammatory ligands such as S100/calgran-ulins, amphoterin, and amyloid-β-peptide (Ramasamy et al. 2005). Levels of RAGE in the lung are quite high, and expression has been demonstrated on the basolateral surfaces of alveolar epithelial type I cells (Shirasawa et al. 2004). RAGE has been recognized for its role in several chronic diseases, such as diabetes, atherosclerosis, coronary artery disease, and lung cancer (Bierhaus et al. 2005; Falcone et al. 2005; Hofmann et al. 2004). In addition to the membrane-associated receptor, a soluble form of the receptor has been identified in mice and humans [soluble RAGE (sRAGE)] (Hanford et al. 2004). The presence of this soluble receptor is thought to provide a binding site for sequestration of ligands, thus reducing the cellular effects of binding to membrane-associated RAGE (Bierhaus et al. 2005). Plasma levels of sRAGE have been found to be decreased in association with the presence of coronary artery disease in nondiabetic men (Falcone et al. 2005). Soluble RAGE is also decreased in patients with rheumatoid arthritis, indicating deficient inflammatory control (Pullerits et al. 2005). In arthritic patients, addition of sRAGE attenuated joint inflammation and expression of inflammatory and tissue-destructive mediators (Moser et al. 2005). In a diabetic mouse model, application of sRAGE reduced inflammation, reduced skin wound healing times, and decreased the protein and enzymatic activity levels of matrix metalloproteinase-9 (MMP-9) (Goova et al. 2001). Based on these known actions of RAGE, alterations in levels of expression of this protein could contribute to As-induced diseases such as chronic lung disease, cancer, peripheral vascular disease, and diabetes.

Several mechanisms can potentially explain the As-induced reductions in RAGE levels in the lavage and sputum samples. Arsenic could be directly binding to the sRAGE, inhibiting recognition by the antibodies used in the ELISA. This does not seem likely because adding As^3+^ or MMA^III^ to samples containing recombinant RAGE did not affect the measured levels. Arsenic could be altering the levels of compounds that bind to the receptor or the levels of proteases that degrade the receptor. Both would be expected to reduce the measurable levels in the sputum samples. Again, this seems unlikely because addition of recombinant RAGE to sputum samples did not affect measured levels compared with controls. Finally, As may change *RAGE* gene expression by altering promoter region methylation or by affecting transcriptional regulators of RAGE (Li and Schmidt 1997; Tohgi et al. 1999).

We have previously reported from the same human population that As exposure is also associated with a significant increase in the MMP-9/TIMP1 (tissue inhibitor of metalloproteinase) ratio, indicative of ongoing inflammation (Josyula et al. 2006). Our analysis did not show a correlation between MMP-9/TIMP1 and RAGE (data not shown). However activation of cellular RAGE has been shown to increase MMP-9 expression (Goova et al. 2001).

There are several other proteins of note that we have identified as being altered after As exposures. These include GST-omega-1, enolase-1, apolipoprotein A-1, and peroxi-redoxin-6. GST-omega-1 has been shown to be an As reductase, having the ability to reduce As^V^ species to the more toxic As^III^ species (Zakharyan et al. 2001). Although it would be expected to be a cytoplasmic protein, other GSTs have been identified in BALF by proteomic techniques (Wattiez and Falmagne 2005). Increased levels of enolase-1 and decreased levels of apolipoprotein A-1 have been previously described as markers of lung cancer (Rutters et al. 2006). The presence of two distinct spots for enolase-1 indicates that posttranslational modifications may be present, as has been reported (Tanaka et al. 1995). Peroxiredoxin-6 is highly expressed in the lung and is localized predominantly in alveolar epithelial type II cells and in Clara cells. It has a dual function as a glutathione peroxidase and as a phospholipase A2 and is induced by oxidative stress (Kim et al. 2003). Alterations in peroxiredoxin-6 suggests that As is playing a role by inducing oxidative stress. In addition, both RAGE and peroxiredoxin-6 are highly expressed in the distal airways and in alveoli. This also suggests that the distal lung may be a site of action for As. Proteomics analysis has shown differences in these proteins in mice, but we have not yet analyzed these proteins in humans. We expect that a single biomarker protein will not be sufficient to fully describe the response to As exposure. Rather, a panel of proteins will be required to more comprehensively determine the effects of As exposure.

Although the majority of analyses in human diseases have been done using ELISA techniques for specific proteins, the use of proteomics as a tool to search for disease markers in BALF has previously been proposed (Wattiez and Falmagne 2005; Wattiez et al. 2003). We recognize that many of the proteins seen in these proteomic analyses are also seen in our experiments. This means that quantitative analysis of levels of expression for these proteins must be performed to evaluate their usefulness as good biomarkers. However, RAGE has not been previously identified as a potential biomarker in lung-lining fluid, and thus, our finding of alterations in RAGE in animal models and humans in response to As is unique and supports its identification as a pulmonary biomarker of As exposure or effect.

## Conclusion

We used a proteomics approach to evaluate As-induced changes in BAL proteins in mice. Six proteins were identified as potential bio-markers. Based on these findings, we tested one of the proteins, RAGE, and found that it was reduced in induced sputum from humans exposed to As in their drinking water. Using a proteomics approach permits a relatively unbiased evaluation of numerous proteins simultaneously, making it possible to detect molecular changes associated with low-dose environmental exposure before the development of clinically apparent disease. We plan to use patterns of changes in multiple proteins measured simultaneously to define modes or mechanisms of action that can be compared among toxicants and compared among biological systems including humans, human cell lines, and rodents.

## Figures and Tables

**Table 1:** Proteins identified as being differentially expressed following 2-D SDS-PAGE/isoelectric focusing separation of BAL proteins from control (*n* = 3) and As-treated (50 ppb; *n* = 3) animals.

Gel, spot no.	Identified protein	Accession no.^a^	No. of peptides	Percent coverage
Control				
1	RAGE	AAH61182	4	9.0
3	GST omega-1	AAH85165	2	7.9
10	Contraspin	CAA40106	2	8.4
13	Apolipoprotein A-IV	AAH50149	3	9.6
14	Apolipoprotein A-I	AAB58426	4	17.5
50 ppb As				
6	Peroxiredoxin-6	NP_031479	5	28.6
16	Enolase 1	AAH39179	5	16.8
17	Enolase 1	AAH39179	2	6.1

Spots differing in intensity and appearance were cut out of the gels, digested, and identified using MS. These qualitative data give possible candidates for further analysis by quantitative methods.

a Accession numbers from NCBI (2005).

**Table 2:** MUDPIT analysis of proteins in BALF from control mice and mice exposed to 10 or 50 ppb As^3+^ in their drinking water for 4 weeks.

Present						
Control	10 ppb	50 ppb	Identified protein	Accession no.^a^	No. of peptides	Percent coverage
X	X	X	Alpha-2-HS-glycoprotein	AAH12678	3	18.0
X	X	X	Apolipoprotein A-I^b^	AAH19837	4	16.8
X	X	X	Carboxylesterase precursor	P23953	2	5.2
X	X	X	Clara cell protein 10	AAH27518	1	14.3
X	X	X	Contraspin (Serpina 3k)^b^	CAA40106	10	33.0
X	X	X	GST omega 1^b^	AAH85165	2	10.0
X	X	X	Hemopexin	AAH19901	9	27.2
X	X	X	Kininogen 1	AAH18158	1	1.4
X	X	X	Plasminogen	AAH57186	3	6.2
X	X	X	Pregnancy zone protein	AAH57983	3	2.5
X	X	X	RAGE^b^	AAH61182	3	8.2
X	X	X	Serpina 1a	AAH37007	7	23.5
X	X	X	Serpina 1b	AAH25445	6	21.6
X	X	X	Serpina 1d	NP_033272	7	25.4
X	X	X	Serpina 1e	AAH61176	5	17.2
X	X	X	Transferrin	AAH22986	24	41.0
X	X	X	Vtamin D-binding protein	AAH51395	4	14.2
	X	X	Aldehyde dehydrogenase 1A7	AAH46315	2	6.2
	X	X	Complement component 3	AAH43338	5	4.9
	X	X	Creatine kinase, muscle	P07310	2	7.1
	X	X	Gelsolin, cytosolic	AAH23143	1	1.5
	X	X	Igh-6	AAH99618	1	2.6
	X	X	Inter-alpha trypsin inhibitor, heavy chain 2	AAH34341	2	3.5
	X	X	Leucine-rich alpha-2-glycoprotein	AAH92077	2	7.9
	X	X	Mast cell protease 7	AAH11328	1	28.3
	X	X	Murinoglobulin 1	AAH51037	2	1.8
	X	X	Peroxiredoxin-6^b^	AAH13489	1	4.5
	X	X	Serpinc1(antithrombin)	AAH33377	1	2.2
	X	X	Transthyretin	AAH86926	3	34.7
		X	EGF-binding protein type A	NP_034244	2	7.7
		X	EGF-binding protein type B	NP_034774	4	23.0
		X	Kallikrein 1-related peptidase b16	AAH12237	2	11.9
		X	Glyceraldehyde-3-phosphate dehydrogenase	AAH92264	2	8.4
		X	Kallikrein 27	NP_064664	2	11.0
		X	Myoglobin	AAH25172	2	20.8
		X	Nerve growth factor, gamma	CAA25930	2	11.8
		X	Parotid secretory protein	AAH53336	2	12.8
		X	Pyruvate kinase 3	AAH94663	2	5.8
X	X		Aldehyde dehydrogenase 1A1	AAH44729	1	2.9
X	X		Apolipoprotein A-II	AAH31786	1	9.8
X	X		Apolipoprotein A-IV^b^	AAH50149	2	8.1
X	X		Apolipoprotein H	AAH53338	1	4.1
X	X		Carboxylesterase 3	Q8VCT4	1	2.5
X	X		Fetub protein	AAH18341	2	4.9

Two animals per dose were analyzed. Only proteins in which multiple peptides were identified or in which single peptides were identified in multiple animals were considered positives; albumin and hemoglobins were excluded.

a Accession numbers from NCBI (2005).

b Proteins were also identified using 2-D gel analysis.

**Table 3:** Population data by town.

Characteristic	Ajo	Tucson	*p*-Value^a^
No. of subjects	34	23	
Female	21 (62)	13 (57)	
Age (years)			
All	63.2 ± 15.6	65.4 ± 14.4	
< 60	12 (35)	9 (39)	
61–80	16 (47)	10 (44)	
> 80	6 (18)	4 (17)	
Ethnicity			
Hispanic	13 (38)	6 (26)	
White non-Hispanic	21 (62)	17 (74)	
Personal history			
Past smoking	15 (44)	11 (52)	
Preexisting illness			
Kidney	6 (18)	4 (17)	
Ever asthma	12 (35)	13 (56)	
Ever diabetes	7 (21)	2 (9)	
Water As levels (*n*)	29	21	
Total water As (μg/L)	20.2 ± 3.6	4.0 ± 2.4	< 0.001
Urine As levels (*n*)	34	22	
Total urinary As (μg/)	27.4 ± 18.5	15.8 ± 15.7	0.019
Total urinary inorganic As (μg/L)	28.2 ± 20.5	13.0 ± 13.7	0.004
Urinary As^3+^ + As^5+^ (μg/L)	8.6 ± 9.0	6.0 ± 10.1	0.328
Total urinary MMA^V^ (μg/L)	2.9 ± 3.9	1.1 ± 1.2	0.003^b^
Total urinary DMA (μg/L)	16.7 ± 11.7	5.8 ± 4.8	< 0.001^b^
Urinary MMA^V^/(As^3+^ + As^5+^)	0.73 ± 0.67	2.12 ± 3.79	0.572^b^
Creatinine-adjusted urinary As (μg/g)^c^	32.3 ± 13.3	16.1 ± 10.3	< 0.001
Sputum RAGE levels (*n*)	34	23	
RAGE (pg/mL)	5,473 ± 25,824	1,336 ± 1,260	0.662^b^
RAGE excluding regression outliers (pg/mL)	1,192 ± 962	1,396 ± 1,254	0.844^b^

Values shown are number (%) or mean ± SD, except where indicated. *p*-Value < 0.05 is statistically significant.

a Compared using independent sample *t*-test with normally distributed test variable, unless specified otherwise.

b The comparisons were performed using two-sample *t*-test with log-normalized variable data.

c Valid values: *n* = 12 for Ajo and *n* = 19 for Tucson.

**Table 4:** Regression models and β coefficients by individuals.

No.	Adjusted *R*^2^	Outcome variable	Predictor variables	β coefficient	*p*-Value
47	0.210	ln RAGE^a^	Town	0.011	0.973
			Diabetes	0.761	0.079
			BMI	0.091	0.002
			Total urinary inorganic As	−0.021	0.016

a Excludes regression outliers. *p*-Value < 0.05 is significant.
